# Single-crystal structure determination of psilocybin after dehydration

**DOI:** 10.1107/S2053229626007692

**Published:** 2026-08-07

**Authors:** Sonya K. Emmett, Lee Cameron, Mark Hestermann, Darren Holland, Simon Kessler, Luke McFarlane, Yit-Heng Chooi, Stephen A. Moggach

**Affiliations:** a School of Molecular Sciences, The University of Western Australia, Perth, WA 6009, Australia; b Natural MedTech, Melbourne, VIC 3146, Australia; University of Strathclyde, United Kingdom

**Keywords:** crystal structure, psilocybin, phase elucidation, anhydrous, phosphate, psychoactive compound, polymorphs

## Abstract

The crystal structure of the known low-tem­per­a­ture anhydrous phase of psilocy­bin {3-[2-(di­methyl­amino)­eth­yl]-1*H*-indol-4-yl di­hy­dro­gen phosphate, C_12_H_17_N_2_O_4_P} is reported. While the structure was previously characterized using powder X-ray diffraction, this study describes the first determination *via* single-crystal X-ray diffraction. High-resolution structural models were ob­tained both before and after *in situ* dehydration, allowing for a direct com­parison of the lattice response to water loss.

## Abstract 2

The structures of the hydrous and first anhydrous phases of crystalline psilocybin have been determined using single-crystal X-ray diffraction.

## Introduction

**Figure 1:**
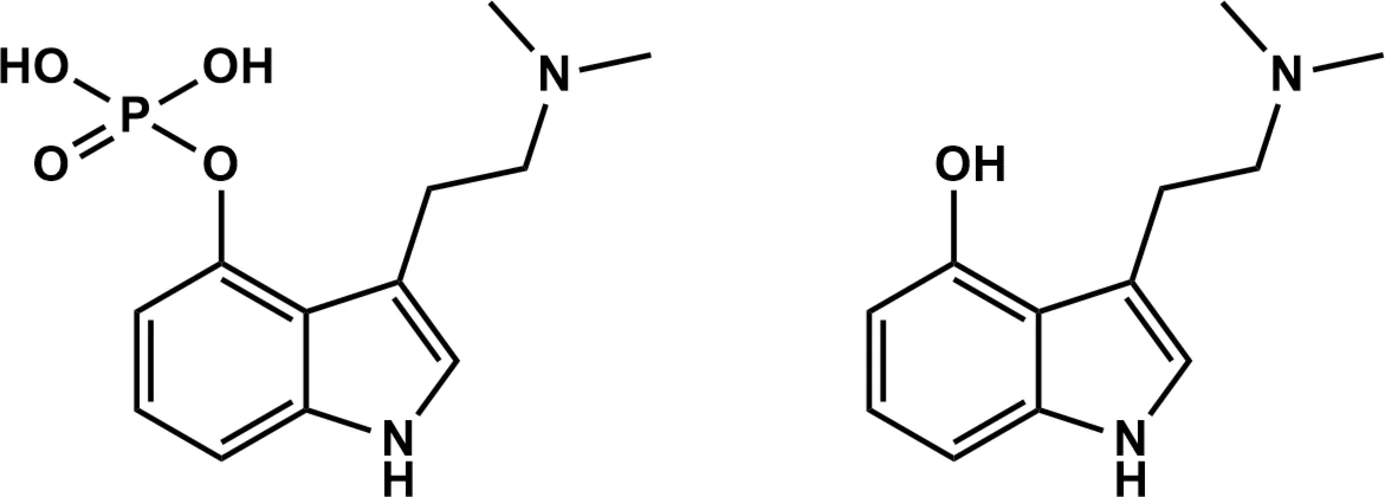
Chemical structure of psilocybin (left) and psilocin (right).

Found as a natural product in various mushrooms around the world, *psilocybin* is a phospho­rylated tryptamine derivative that undergoes de­phospho­rylation within the human body to produce the psychoactive com­pound *psilocin* (Fig. 1 ▸) (T­ylš *et al.*, 2014 ▸). The therapeutic properties of psilocybin have attracted renewed inter­est in the medical field in the last two decades due to an observed efficacy in the treatment of psychiatric and substance abuse disorders, including post-traumatic stress disorder, treatment resistant depression, anxiety and addiction to nicotine and alcohol (Bogenschutz *et al.*, 2015 ▸; Johnson *et al.*, 2014 ▸).

Psilocybin has been reported previously with two anhydrous phases, one trihydrate, six solvates and one cocrystal (Table 1 ▸) (Sherwood *et al.*, 2022 ▸). After the drug’s first synthesis in 1958 (Hofmann *et al.*, 1958 ▸), the anhydrous form of psilocybin was first reported from separately dried trihydrate and methano­late solvates in 1976, and confirmed with IR spectroscopy and differential scanning calorimetry (DSC) (Kuhnert-Brandstätter & Heindl, 1976 ▸). The hygroscopic nature of psilocybin was also observed in this study, and the authors noted that transitions between the hydrous and anhydrous forms could occur depending on environmental factors such as humidity and tem­per­a­ture.

The first and second anhydrous phases of psilocybin were noted in 2018 under a patent application by Compass Pathways (Londesbrough *et al.*, 2019 ▸). These were labelled as *Polymorph A′* (the first lower-tem­per­a­ture anhydrate) and *Polymorph B* (a second higher-tem­per­a­ture anhydrate), and were reported using powder X-ray diffraction (PXRD). A third patent was lodged under the label *Polymorph A* as a ‘novel isostructural variant’. In 2022, *Polymorph A* was shown by Sherwood *et al.* (2022 ▸) to be a mixed material of *Polymorph A′* and *Polymorph B* rather than a new phase.

While structure solution with single-crystal X-ray diffraction (SCXRD) has been successfully carried out for many of psilocybin’s solvates, poor crystal quality or small crystal size due to mechanical fracturing during dehydration is cited as a significant issue preventing the same being done for anhydrous psilocybin. The structures of *Polymorph A′* and *Polymorph B* were solved at 295 K from high-resolution PXRD patterns in 2022, but SCXRD data of either psilocybin anhydrate phase have been unavailable (Sherwood *et al.*, 2022 ▸). In the present study, psilocybin obtained *via* biosynthetic production using an engineered yeast system was used as the starting material for crystallization and structure analysis, providing access to material suitable for single-crystal analysis. With this in mind, herein we describe the dehydration protocol and first SCXRD structure of the anhydrous *Polymorph A′* form of psilocybin. *Polymorph A′* is the crystalline anhydrate that first forms during the drying process, and changes in partial trace amounts into *Polymorph B* upon drying at ambient pressures above 38–40 °C (Kargbo *et al.*, 2022 ▸; Greenan *et al.*, 2020 ▸). *Polymorph A′* is converted to the higher-tem­per­a­ture phase in totality above 160 °C.

## Experimental

### Sample preparation

**Figure 2:**
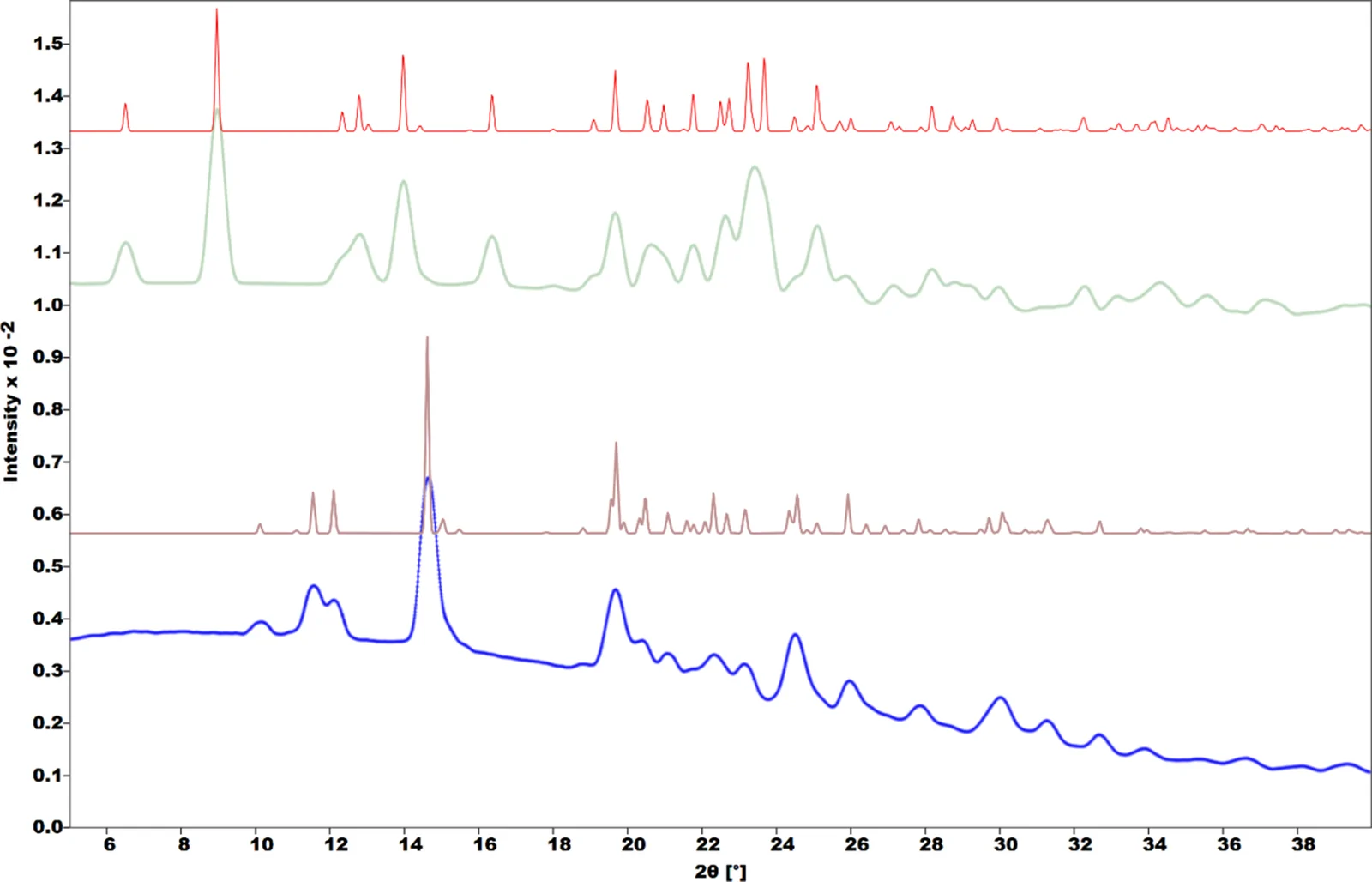
Comparison of the PXRD patterns for psilocybin trihydrate and *Polymorph A′*. The experimental patterns (green for the trihydrate and blue for *Polymorph A′*) are shown alongside their respective calculated patterns derived from SCXRD data (red for the trihydrate and grey for *Polymorph A′*) for structural validation.

A two-step drying process was used to obtain crystals of only *Polymorph A′*, following the method outlined by Sherwood *et al.* (2022 ▸). A sample of psilocybin was provided by Natural MedTech and recrystallized from water at 60 °C, then dried under vacuum for 24 h at tem­per­a­tures between 35 and 45 °C. A sub­sample of these hydrated crystals was removed for analysis, and the remaining crystals were then dried under vacuum for a further 24 h in the tem­per­a­ture range 50–60 °C. PXRD data were collected under ambient tem­per­a­ture conditions on both the hydrated sub­sample and the dehydrated sample to screen the phase com­position of the bulk material. PXRD data were collected on sub- to micron-sized crystals on a Synergy-S dif­frac­tom­eter in transmission geometry, which confirmed the first-step sub­sample and final two-step product as predominantly psilocybin trihydrate and *Polymorph A′*, respectively (Fig. 2 ▸).

### SCXRD structure solution and refinement details

The particle size distribution of the original sample was significant, with sub-to micron-sized crystals, as well as several crystallites of 50 µm and bigger. SCXRD data were collected on these larger crystals from the bulk samples. Data collection was conducted at 100 K on the recrystallized hydrate phase and on a separate crystal from the post heated and dried sample. Relevant crystallographic information is provided in Table 2 ▸.

On initial inspection, anhydrous *Polymorph A′* appeared subtely different to the reported anhydrous phase of Sherwood *et al.* (2022 ▸) due to a transformation of the unit-cell dimensions. In order to com­pare with the published structure [Cambridge Structural Database (CSD; Groom *et al.*, 2016 ▸) refcode TAVZID], we transformed the unit-cell dimensions by applying the following matrix: 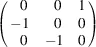


## Results and discussion

**Figure 3:**
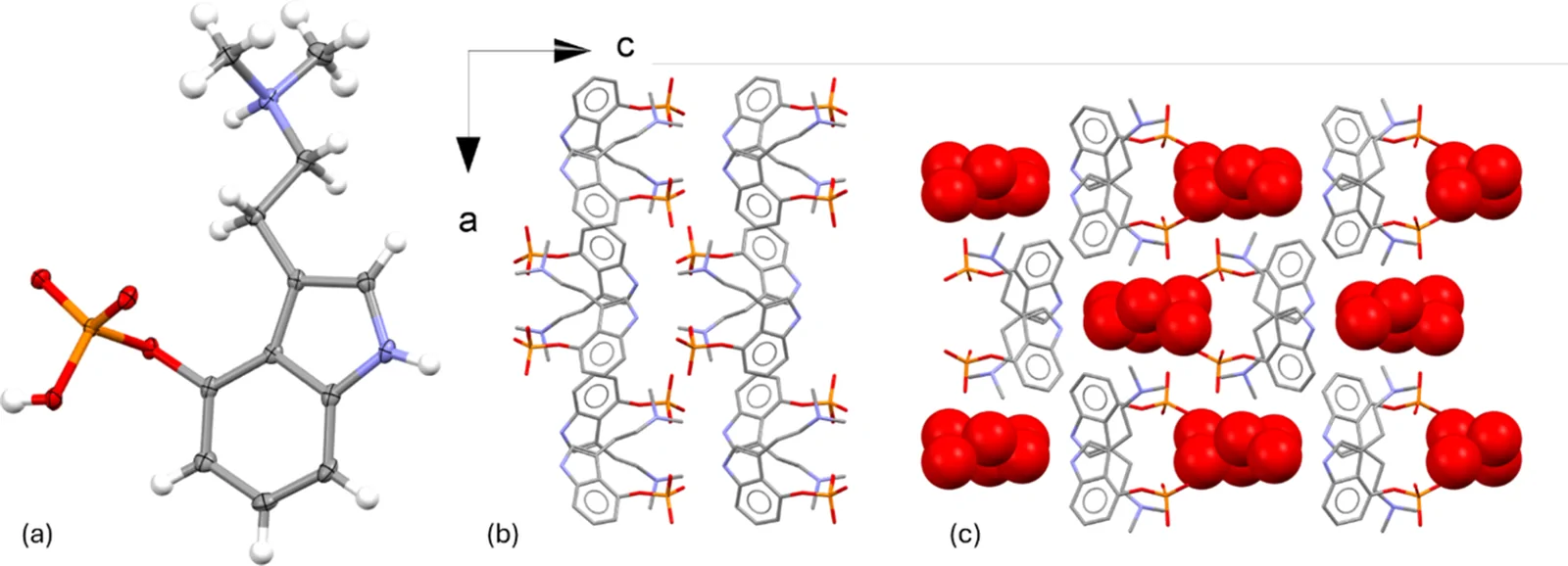
(*a*) The mol­ecular connectivity of psilocybin (taken from the trihydrate crystal structure), with displacement ellipsoids drawn at the 50% probability level. Colour code: C grey, H white, N blue, O red and P orange. Crystal packing of (*b*) the anhydrous *Polymorph A′* and (*c*) the trihydrate form, both viewed down the *b* axis. Mol­ecules are shown in stick representation, with the exception of the water O atoms in the trihydrate, which are rendered as red space-filling spheres to highlight their positions within the channels. Note the significant contraction of the unit cell along the *c* axis upon dehydration, corresponding to the collapse of the lattice into the anhydrous phase.

During the first step, psilocybin trihydrate crystallized in the ortho­rhom­bic space group *Pbca*, with water-filled channels parallel to the *b* axis stabilized by hy­dro­gen bonding to the phosphate moieties (Fig. 3 ▸ and Fig. S1 in the supporting information). During the initial stage of dehydration, thermal or vacuum driving forces induce the rapid loss of water mol­ecules occupying the 22.7% void volume (729.7 Å^3^) within the one-dimensional channels running parallel to the *b* axis. This loss breaks the critical host–guest hy­dro­gen bonds stabilizing the phosphate moieties, leaving unsupported cylindrical cavities within the lattice. Deprived of solvent support, these channels collapse inward along the transverse crystal directions, causing a large contraction along the *c*-axis direction [with a reduction from 27.0980 (10) to 17.466 (5) Å]. To optimize space-filling in the absence of channel water, neighbouring stacks of zwitterionic psilocybin mol­ecules undergo a co-operative shear-like sliding motion past one another, closing the macroscopic voids. When water evacuation occurs rapidly at the crystal exterior, localized channel collapse near the surface can seal off inter­ior channels, creating trapped pockets of high-pressure water vapour. The combined localized strain from both side-chain deformation and trapped vapour pressure ultimately leads to micro-cracking and crystal fracturing, explaining the mechanically destructive nature of the dehydration transition. This structural reorganization drives a specific rearrangement of the hy­dro­gen-bonding net­work. While the phosphate groups remain paired as a dimer and retain a direct hy­dro­gen bond from the protonated di­methyl­ammonium group in both phases, the loss of solvent causes the phosphate group to forfeit two hy­dro­gen bonds with channel water. To com­pensate, the collapse allows the phosphate group to form a new direct N—H⋯O hy­dro­gen bond with the indole N—H group – a contact that was strictly water-mediated prior to dehydration.

Comparison of this experimental trihydrate model with the previously reported structure reveals negligible structural dis­parity. A three-dimensional mol­ecular overlay yields a root-mean-square deviation (RMSD) of 0.0121 Å and a maximum atomic displacement of 0.0280 Å, con­firming that the atomic positions and mol­ecular conformation in our structure are virtually identical to the published reference data (Arlin *et al.*, 2021 ▸).

Psilocybin anhydrate *Polymorph A′* crystallized in the ortho­rhom­bic space group *Pbca*, with a unit-cell volume of 2592.1 (11) Å^3^ [Tables 2 ▸ and 3 ▸, and Fig. 3 ▸(*b*)]. The anhydrous psilocybin structure outlined herein is structurally analogous to Sherwood’s prior solution, however, it has been re-indexed conventionally using the directionality insights provided by SCXRD. The indexing we use also makes it easier to com­pare the trihydrate and anhydrous *Polymorph A′* forms. The unit-cell dimensions we determined are marginally smaller, which is unsurprising considering that the data were collected at 100 K, whereas the original PXRD data were collected at room tem­per­a­ture (298 K).

Data for the anhydrous *Polymorph A′* could only be collected to 1 Å resolution without prohibitively long collection times. This is expected, as the loss of water results in a 19.35% reduction in the unit-cell volume. Notably, the successful collection of large intact crystals post-dehydration was a highlight of this study, as the rapid evacuation of water typically causes significant mechanical fracturing.

To qu­anti­tatively com­pare our single-crystal structure of anhydrous *Polymorph A′* with the literature, structural overlays were performed using *Mercury* (Macrae *et al.*, 2020 ▸). Our experimental SCXRD structure shows outstanding agreement with both the experimental powder structure reported by Sherwood and co-workers (RMSD = 0.082 Å; mol­ecular overlay = 0.042 Å) and a VASP (Vienna Ab initio Simulation Package)-optimized DFT (density functional theory) model (RMSD = 0.090 Å; mol­ecular overlay = 0.039 Å), which was reported in the same article. This remarkably low RMSD confirms that the single-crystal structure determination accurately captures the true anhydrous geometry. The *Mogul* (Bruno *et al.*, 2004 ▸) intra­molecular geometry check highlights notable conformational differences in the di­methyl­amino­ethyl side chain in the hydrated and anhydrous forms. Notably, in the trihydrate, the C—N—N—C torsion angles adopt expected values of ap­prox­i­mately ≈65° and ≈−70°, corresponding to standard low-energy *gauche* preferences. In contrast, the experimental torsion angle in the anhydrous phase measures 86.8 and −149.7°, and falls directly into the low-frequency local minimum between these two main energy wells, further highlighting the torsional strain accommodated in the anhydrous crystal packing.

## Conclusion

In summary, this study demonstrates that the dehydration of psilocybin trihydrate can be achieved through a controlled two-step vacuum-drying protocol. Despite the significant 19.35% reduction in unit-cell volume – driven by the collapse of water-filled channels – we have successfully reported the first single-crystal X-ray diffraction structure of an anhydrous psilocybin phase, denoted *Polymorph A′*. While dehydration is often considered a mechanically destructive process, this work confirms that large intact single crystals can be recovered and analyzed, overcoming previous limitations in structure characterization. These findings provide critical insights into the structural response of psilocybin to water loss and establish a foundation for further investigation into its various solid-state forms.

## Supplementary Material

Crystal structure: contains datablock(s) ps1_recryst_dehydrated_100k_auto, ps1_100k_hydrate_recryst_auto, global. DOI: 10.1107/S2053229626007692/vp3057sup1.cif

Structure factors: contains datablock(s) ps1_recryst_dehydrated_100k_auto. DOI: 10.1107/S2053229626007692/vp3057ps1_recryst_dehydrated_100k_autosup2.hkl

Structure factors: contains datablock(s) ps1_100k_hydrate_recryst_auto. DOI: 10.1107/S2053229626007692/vp3057ps1_100k_hydrate_recryst_autosup3.hkl

Single-crystal X-ray diffraction structures of psilocybin showing the dehydrated form alongside the trihydrate phase. DOI: 10.1107/S2053229626007692/vp3057sup4.pdf

CCDC references: 2562278, 2562279

## Figures and Tables

**Table 1:** Reported phases of anhydrous psilocybin and precursor solvates, and method of characterization

	SCXRD	PXRD	DSC	IR
Methanol solvate (2:1 *v*/*v*)	Weber & Petcher (1974 ▸)	Greenan *et al.* (2020 ▸)	Kuhnert-Brandstätter & Heindl (1976 ▸)	Kuhnert-Brandstätter & Heindl (1976 ▸)
Trihydrate	Greenan *et al.* (2020 ▸)	Greenan *et al.* (2020 ▸) and Sherwood *et al.* (2022 ▸)	Kuhnert-Brandstätter & Heindl (1976 ▸)	Kuhnert-Brandstätter & Heindl (1976 ▸)
First anhydrate/*Polymorph A′*	–	Greenan *et al.* (2020 ▸)†	Kuhnert-Brandstätter & Heindl (1976 ▸)	Hofmann *et al.* (1958 ▸) and Kuhnert-Brandstätter & Heindl (1976 ▸)
Second anhydrate/*Polymorph B*	–	Greenan *et al.* (2020 ▸), Londesbrough *et al.* (2019 ▸), Sherwood *et al.* (2022 ▸) and Kargbo *et al.* (2022 ▸)	Greenan *et al.* (2020 ▸)	–

† High-resolution PXRD was used to generate a structure solution.

**Table 2:** Experimental details For both determionations: orthorhombic, *Pbca*, *Z* = 8. Experiments were carried out at 100 K with Cu *K*α radiation using a Rigaku XtaLAB Synergy diffrac­tometer with a HyPix detector. Absorption was corrected for by multi-scan methods (*CrysAlis PRO*; Rigaku OD, 2024 ▸). H-atom parameters were constrained.

	Dehydrated psilocybin	Psilocybin trihydrate
Crystal data		
Chemical formula	C_12_H_17_N_2_O_4_P	C_12_H_17_N_2_O_4_P·3H_2_O
*M*_r_	284.24	338.29
*a*, *b*, *c* (Å)	15.920 (3), 9.322 (3), 17.466 (5)	14.3405 (6), 8.2707 (3), 27.098 (1)
*V* (Å^3^)	2592.1 (11)	3214.0 (2)
μ (mm^−1^)	2.02	1.85
Crystal size (mm)	0.23 × 0.15 × 0.11	0.13 × 0.05 × 0.04

Data collection		
*T*_min_, *T*_max_	0.210, 1.000	0.615, 1.000
No. of measured, independent and observed [*I* > 2σ(*I*)] reflections	4440, 1013, 486	12268, 3256, 2382
*R*_int_	0.121	0.091
θ_max_ (°)	45.0	79.3
(sin θ/λ)_max_ (Å^−1^)	0.458	0.637

Refinement		
*R*[*F*^2^ > 2σ(*F*^2^)], *wR*(*F*^2^), *S*	0.082, 0.280, 1.04	0.052, 0.120, 1.04
No. of reflections	1013	3256
No. of parameters	176	211
Δρ_max_, Δρ_min_ (e Å^−3^)	0.32, −0.39	0.36, −0.48

Computer programs: *CrysAlis PRO* (Rigaku OD, 2024 ▸), *SHELXT2018* (Sheldrick, 2015*a* ▸), *SHELXL2019* (Sheldrick, 2015*b* ▸) and *OLEX2* (Dolomanov *et al.*, 2009 ▸).

**Table 3:** Unit-cell dimensions for *Polymorph A′* from published powder (PXRD) and our own single-crystal data (SCXRD), transformed for com­parison

	*Polymorph A′* (PXRD)	*Polymorph A′* (SCXRD)
CCDC refcode	TAVZID	n/a
Temperature (K)	295	100
*a* (Å)	17.4979 (2)	17.466 (5)
*b* (Å)	16.0914 (1)	15.920 (3)
*c* (Å)	9.34815 (7)	9.322 (3)
Volume (Å^3^)	2632.11 (3)	2592.1 (11)
