# Supplementary material for: Single-crystal structure determination of psilocybin after dehydration
Source: Acta Crystallogr C Struct Chem. 2026 Aug 7;82(Pt 9):542–6. doi: 10.1107/S2053229626007692 (PMC13543281; doi:10.1107/S2053229626007692)
Supplement: Supplementary file 1 [file c-82-00542-sup4.pdf]

STRUCTURAL CHEMISTRY

Volume 82 (2026)

Supporting information for article:

Single-crystal structure determination of psilocybin after dehydration

Sonya K. Emmett, Lee Cameron, Mark Hestermann, Darren Holland, Simon Kessler, Luke McFarlane, Yit-Heng Chooi and Stephen A. Moggach

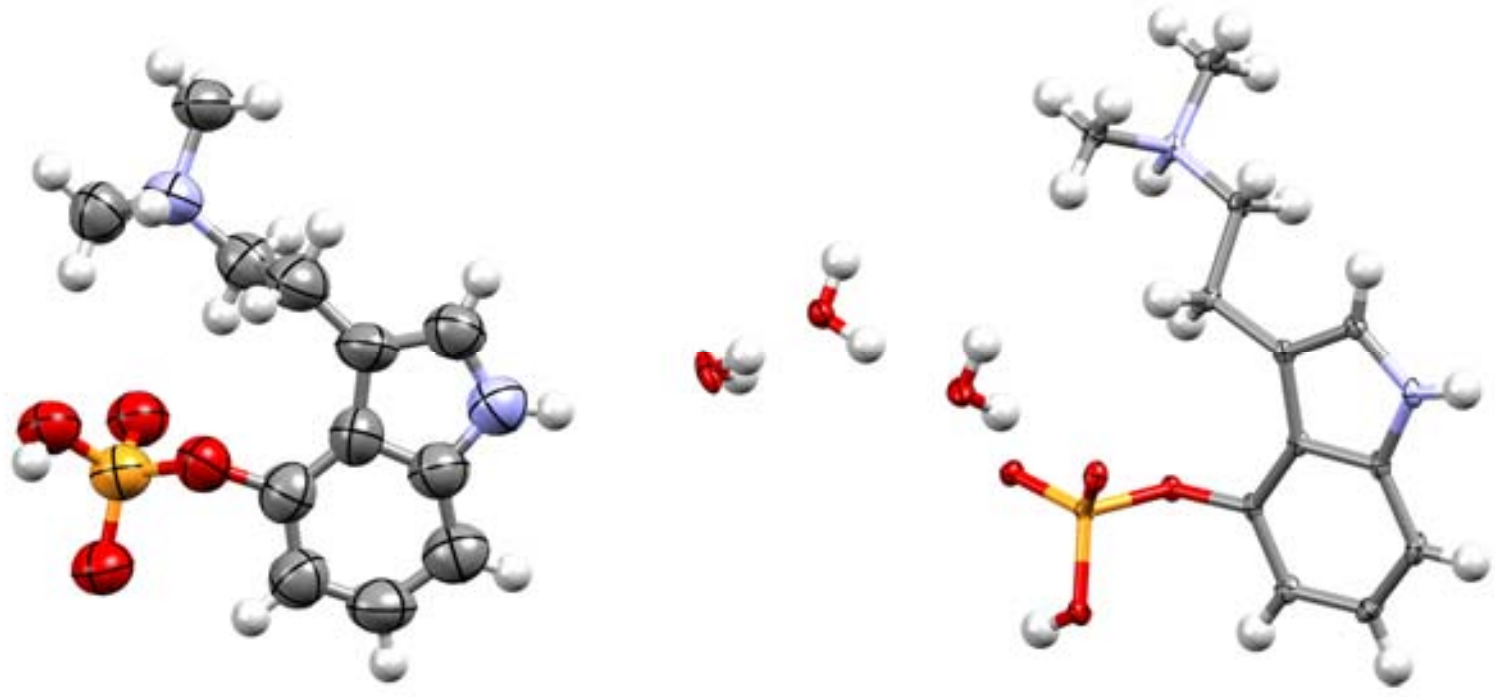

Fig. S1. Single-crystal X-ray diffraction structures of psilocybin showing the dehydrated form, Polymorph A′ (left), alongside the trihydrate phase with associated solvent water molecules (right). Thermal ellipsoids are drawn at the 30% probability level. Hydrogen atoms are represented as fixed-size spheres with a radius of 0.3 Å. Atom colour scheme: carbon (grey), hydrogen (white), nitrogen (blue), oxygen (red), and phosphorus (orange).
